# Effect of the Tea Tree Oil on Growth Performance, Meat Quality, Serum Biochemical Indices, and Antioxidant Capacity in Finishing Pigs

**DOI:** 10.3389/fvets.2022.916625

**Published:** 2022-06-24

**Authors:** Tianyu Yang, Feifei Feng, Kang Zhan, Xiaoyu Ma, Maocheng Jiang, Osmond Datsomor, Xinyu Zhu, Yongjiu Huo, Guoqi Zhao

**Affiliations:** ^1^Institute of Animal Culture Collection and Application, College of Animal Science and Technology, Yangzhou University, Yangzhou, China; ^2^Wuxi Chenfang Biotechnology Co., Ltd., Wuxi, China

**Keywords:** tea tree oil, finishing pigs, growth performance, meat quality, serum biochemical indices, antioxidant capacity

## Abstract

The increased use of antibiotics continues to pose a threat to public health because of the increasing concern of antibiotic residue. Tea tree oil (TTO) is an extract of the Australian plant *Melaleuca alternifolia* with anti-inflammatory and antioxidant properties. However, there is little information on TTO supplementation in the diet of finishing pigs. Hence, the present study aimed to investigate the effect of TTO supplemented diets on the growth performance, meat quality, serum biochemical indices, and antioxidant capacity of the finishing pigs. Our results showed that TTO supplementation increased (*P* < 0.05) the mRNA expression of insulin-like growth factors -I (IGFs-I), growth acceleration hormone (GH), and heart fatty acid-binding protein (H-FABP), while the mRNA expression of myostatin gene (MSTN), and calpain-1 (CAST) decreased by the TTO supplementation, compared with the control group. In addition, TTO supplementation increased (*P* < 0.05) serum alkaline phosphatase (ALP), immunoglobulin G (IgG), and IgM levels but decreased (*P* < 0.05) serum aspartate transaminase (AST) concentration, relative to the control group. In addition, we found that the live weight and intramuscular fat enhanced (*P* < 0.05) significantly, and muscle pH 24 min value, cooking loss, and shear force decreased (*P* < 0.05) dramatically in the TTO group. The TTO supplementation increased (*P* < 0.05) C18:2n6t concentration and decreased (*P* < 0.05) C12:0 and C16:0 concentration, relative to the control group. Dietary supplementation with TTO decreased (*P* < 0.05) malondialdehyde (MDA) and increased (*P* < 0.05) glutathione peroxidase (GSH-Px) activity in serum. These results indicated that TTO supplementation could improve immunity and antioxidant, carcass traits, the nutritional value of pork, and the antioxidant capacity of finishing pigs. Therefore, TTO has potential positive effects as a feed additive in the pig industry.

## Introduction

Antibiotics have been used in livestock to improve feed efficiency, prevent diseases, and increase animal production since the 1950s (1). In recent decades, with improved living standards, consumers are increasing their demand for quality meat products, which provides a healthy balance of nutrients (2–4). The increased use of antibiotics poses an environmental risk and a threat to public health because of the increasing concern of antibiotic residue (5). Therefore, an effective alternative to antibiotics, especially from plants, in pig production is vital for improving meat quality and human health.

Essential oils extracted from the aromatic plants are a potential alternative to antibiotics (6). Tea tree oil (TTO), an essential oil derived from the Australian plant Melaleuca alternifolia, contains more than one hundred different compounds, mainly monoterpenes and their derivatives. The main components of TTO include terpinene-4-ol, γ-terpinene, α-terpinene, 1,8-cineole, and α-terpineol (7). Zhan et al. (8) demonstrated that a supplement of TTO could alleviate inflammatory response in bovine mammary epithelial cells exposed to *Staphylococcus aureus*. Previous studies have focused on antibacterial activity and anti-inflammatory properties (9). In addition, TTO could activate the antioxidant signaling pathway (10), thus, increasing antioxidant capacity and ameliorating oxidative damage in animals (11). Based on TTO's anti-inflammatory and antioxidant properties, numerous research on TTO have also been investigated in animals. TTO can enhance pigs' growth performance, meat quality, antioxidant status, and anti-inflammatory function (12, 13). Previous research showed that TTO supplementation could improve growth performance, promote liver and thymus development, and facilitate intestinal mucosal immunity by activating the Notch2 signaling pathway in the weaning pigs (14). Since dietary lipids are associated with the emergence of coronary heart disease, the lipid content of pork is another concern for consumers. Although it has been demonstrated that a positive effect exists between essential oils and body lipid metabolism (15, 16), there is minimal information regarding diets supplemented with TTO on finishing pigs. Therefore, in the present study, we investigated the effects of TTO supplementation on the growth performance, meat quality, serum biochemical indices, and antioxidant capacity of finishing pigs.

## Materials and Methods

### Animals and Experimental Design

In total, sixty-four (64) finishing pigs (Duroc × Landrace × Yorkshire) with average initial body weight (BW) of 68.13 ± 0.46 kg were randomly divided into 4 treatment groups. Each treatment group contained 16 pigs raised in 4 pens, each with 4 pigs (2 males and 2 females). The 4 treatment groups included the control treatment (CON, feed with only basal diets) and the low-, middle- and high-level TTO-supplemented diets (LTO, MTO, and HTO, feed with the basal diets supplemented with 100 mg/kg, 200 mg/kg, and 300 mg/kg TTO (net content of TTO), respectively). In addition, the feed supplemented with TTO was formulated every day to reduce TTO oxidation. The experiment was conducted at the Jinzhu Agricultural Development Company, Taicang, Suzhou province, China in June 2016.

The TTO utilized was the Australian tea tree oil powder type provided by the Chen Fang Biotechnology Company, Wuxi, China. The TTO was purified and processed by the company so that the constituents, such as 4-terpineol, were not wholly the same as other TTOs (the details are proprietary to the company). Furthermore, TTO was absorbed in microcrystalline cellulose (the net content of the TTO is 20%). In addition, the effective constituents of the TTO were analyzed by gas chromatography-mass spectrometry (QP2010, SHIMADZU Company, Japan) in our previous study (14).

The composition of the basal diet is illustrated in Table 1. Two samples were analyzed for the measured values, and the mean value was calculated as shown in Table 1. The basal diet (Table 1) was formulated to meet or exceed the nutrient requirements recommended by NRC (1998) nutrient requirements. All the pigs were housed in 16 adjacent pens (1.8 × 4.6 m) equipped with slatted floors in an environmentally controlled facility. The pigs were fed two times a day (at 7:00 am and 04:30 pm) with the prepared diet in feeding troughs and had *ad libitum* access to feed and water. The target room temperature was 25°C. Pig BW was measured initially and at week 8 of the experimental period. Feed consumption was recorded on a pen basis during the experiment. The average daily gain (ADG), average daily feed intake (ADFI), and gain-to-feed ratio (G/F) were calculated (13). The experiment lasted for 56 days. At the end of the experiment, three pens were randomly selected from each treatment, from which two pigs were selected per treatment pen, given a total of 6 pigs (3 males and 3 females) for the collection of sample data.

**Table 1:** Composition and nutrient levels of the basal diets.

**Ingredient g/kg**	**Content**
Corn	670
Soybean meal	250
Wheat bran	40
Pre-mixture^a^	40
Total	1,000
**Nutrition levels, g/kg**	
Digestible energy, MJ/kg	13.36
CP^b^	162.5
Ca^b^	5.8
Total P^b^	3.8
Lys	8.6
Met	2.0
Thr	6.5
Trp	2.8

*^a^The premix provided the following per kg of the diet: Fe (as ferrous sulfate) 80 mg, Cu (as copper sulfate) 15 mg, Zn (as zinc sulfate) 80 mg, Mn (as manganese sulfate) 5 mg, Se (as sodium selenite) 0.1 mg, I (as potassium iodide) 0.1 mg, VA 4 480 IU, VD3 500 IU, VE 20 IU, VK_3_ 2.20 mg, VB_1_ 1.80 mg, VB 2 2.20 mg, VB 6 1.50 mg, VB12 12 μg, folic acid 0.30 mg, biotin 0.05 mg, nicotinic acid 10 mg, and calcium pantothenate 8 mg. ^b^CP, crude protein; Ca, calcium; P, phosphorus; Three samples were analyzed for the analyzed values, and the mean value was calculated and shown*.

### Carcass Traits and Meat Quality

All the pigs at each time point were electrically stunned, exsanguinated, eviscerated, dehaired, and peeled according to standard commercial procedures. Carcass weight was recorded. Then, the longissimus dorsi (LD) muscle samples from the left carcass and the 9th rib of the right carcass were collected and refrigerated at 4 for meat quality measurement. About 100 g of LD samples from the 10th rib of the left carcass was collected and frozen at-20°C and used to measure muscle fatty acids profile, the mRNA expression, and IMF content. To know about the methods of meat quality measurement refer to the previous study (13).

### The MRNA Expression Analysis by Real-Time PCR

The total RNA was isolated from the liver, LD muscle, and back fat using TRIzol (Takara, Code No. RR036A). The RT reaction mixtures contained 1 μg total RNA and 5× PrimeScript RT Master Mix in a final volume of 20 μl. The RT reactions were performed for 15 min at 37°C. Reverse transcriptase was inactivated by heating to 85°C for 5 s. qRT–PCR was performed with an SYBR^®^ Premix Ex Taq^TM^ II Kit (Takara, Code Nos. RR820A and RR420A). The qRT–PCR included an initial denaturation at 95°C for 30 s, followed by 40 cycles at 95°C for 5 s and 60°C for 30 s. The primers used are listed in Table 2. The relative expression of target genes was normalized to that of GAPDH and calculated using the 2^−Δ*ΔCT*^ method.

**Table 2:** Primers for real-time PCR analyses.

**Genes**	**Accession**	**Primer sequence 5^′^-3^′^**	**Product**
	**number**		**length/bp**
GH	NM_213869.1	F: GGCTGTGATGGCTGCAGGCC;	658
		R: CTAGAAGGCACAGCTGCTCTCCACG	
IGF-I	NM_214256.1	F: TCTTCTACTTGGCCCTGTGCTT;	73
		R: GCCCCACAGAGGGTCTCA	
IGF-II	NM_213883.2	F: CCGGACAACTTCCCCAGATA;	71
		R: CGTTGGGCGGACTGCTT	
MSTN	NM_214435.2	F: CCAGAGAGATGACAGCAGTGATG;	113
		R: TTCCTTCCACTTGCATTAGAAGATC	
A-FABP	NM_001002817.1	F: CAGGAAAGTCAAGAGCACCA	227
		R: TCGGGACAATACATCCAACA	
H-FABP	NM_001099931.1	F: GCCAACATGACCAAGCCTACC′	262
		R: CATGGGTGAGTGTCAGGATGAG	
CAST	NM_001001534.1	F: GCGTGCTCATAAAGAAAAAGC	133
		R: TGCAGATACACCAGTAACAG	
CAPN1	NM_001348784.2	F: CCAACAAGGAGGGCGACTT	57
		R: GGGTCCCGGCTTTCTTCTC	
GAPDH	NM_001256799.2	F: TCGGAGTGAACGGATTTG;	219
		R: CCTGGAAGATGGTGATGG	

*F, forward; R, reverse*.

### Serum Biochemical Indices

Total serum levels of immunoglobulin IgG, IgA, and IgM (g/L) were measured by commercially available ELISA kits from the Nanjing Jiancheng Bioengineering Institute (Nanjing, China). Alanine aminotransferase (ALT), aspartate aminotransferase (AST), and alkaline phosphatase (ALP) were analyzed by an automatic biochemical analyzer.

### Measurement of Serum, Liver, and LD Muscle Antioxidant

The malondialdehyde (MDA), total superoxide dismutase (T-SOD) activity, catalase (CAT) activity, and glutathione peroxidase (GSH–PX) activity were measured using diagnostic kits (the Nanjing Jiancheng Bioengineering Institute, Nanjing, Jiangsu, P. R. China) according to the manufacturer's instructions.

### Fatty Acid Composition of Longissimus Muscle

Fatty acid content was determined according to GB/5413.27-2010. A 4 g thick section of longissimus muscle was removed from the center (in the region of the 10th rib) of a boneless pork loin. The sample was trimmed free of all subcutaneous fat and epimysial connective tissue. All the samples were immediately frozen at-80°C until fatty acid analysis.

Longissimus muscles were placed in 30 ml beakers, then placed into vacuum flasks attached to the manifold of a Labconco freeze-dryer (LyoQUEST-55, USA). After freeze-dried, 0.5 g of LD muscle was weighed and placed in a 15 mg spiral glass tube. In total, 5.0 ml toluene and 6.0 ml 10% acetyl chloromethanol were added and the tube was filled with nitrogen to ensure total air removal within the tube. The mixture was homogenized, placed in a water bath at 80°C for 2 h, and vibrated once every 20 min. The mixture was taken out and cooled to room temperature. The cooled liquid was transferred to a 50 ml centrifuge tube, and the centrifuge tube was cleaned with sodium carbonate solution and transferred to a new 50 ml centrifuge tube. The glass tube was shaken and mixed, centrifuged at 5,000 r/min for 5 min, and the supernatant was measured by meteorological chromatograph; equipped with a 100-m capillary column (0.25-mm i.d.; Model 2560 fused-silica capillary column, Supelco Inc., Bellefonte, PA) and helium as the carrier gas at 1 ml/min (1:30 split ratio). The oven temperature was maintained at 140°C for 5 min, increased at 4°C/min to 240°C for 15 min, whereas injector temperatures were maintained at 260°C and detector temperatures were maintained at 280°C.

### Statistical Analysis

Statistical analysis was performed by one-way analysis of variance (ANOVA) followed by the least significant difference (LSD) test for *post-hoc* correction for multiple comparisons of treatment means using the SPSS 16.0 software (SPSS Inc.; Chicago, IL, USA). The *P* values are represented in the figures as follows: statistical significance was set at *P* < 0.05.

## Results

### Growth Performance

The effects of TTO on the growth performance of finishing pigs are shown in the Supplementary Table 1. Compared with the control group, TTO supplementation increased (*P* < 0.05) final weight and the ADG value, while the ratio of F/G were decreased (*P* < 0.05) by TTO addition.

### Relative Gene Expression of Growth Performance of Finishing Pigs

The effect of TTO on the relative gene expression of GH, IGF-I, IGF-II, and MSTN of growth performance is shown in Table 3. Relative to CON pigs, MTO and HTO supplementation increased (*P* < 0.05) the mRNA expression of GH and IGF-I in LD muscle, and the mRNA expression of IGF-I was up-regulating (*P* < 0.05) with TTO supplementation in the liver. In addition, the gene expression of MSTN was decreased (*P* < 0.05) by TTO addition in the liver and LD muscle.

**Table 3:** Effect of TTO on the genes expression of GH, IGF-I, IGF-II, and MSTN of finishing pigs (*n* = 6).

		**Treatment^1^**					
**Sites**	**Symbol**	**CON**	**LTO**	**MTO**	**HTO**	**SEM**	***P*-value**
Liver	GH	1.06	1.02	1.52	1.13	0.09	0.21
	IGF-I	1.03^b^	2.98^a^	4.25^a^	3.06^a^	0.33	0.002
	IGF-II	1.01	0.93	0.93	0.78	0.05	0.47
	MSTN	1.02^a^	0.69^b^	0.73^b^	0.70^b^	0.47	0.03
LD muscle	GH	1.02^b^	1.17^ab^	1.88^a^	1.94^a^	0.15	0.04
	IGF-I	1.00^b^	1.11^b^	1.72^a^	1.24^b^	0.07	<0.001
	IGF-II	1.02	1.29	1.03	0.98	0.66	0.36
	MSTN	1.00^a^	0.72^b^	0.73^b^	0.74^b^	0.04	0.007
Back fat	GH	1.01	1.10	1.32	1.26	0.08	0.48
	IGF-I	1.01	1.12	1.10	1.08	0.07	0.95
	IGF-II	1.01	1.04	1.20	0.96	0.07	0.70

*^a, b^Means in the same row with different superscripts differ significantly for treatment effect. ^1^CON (control), pigs receiving a control diet; LTO (low tea tree oil supplemented treatment), pigs receiving a control diet supplemented with 100 mg/kg tea tree oil; MTO (middle tea tree oil supplemented treatment), pigs receiving a control diet supplemented with 200 mg/kg tea tree oil; HTO (high tea tree oil supplemented treatment), pigs receiving a control diet supplemented with 300 mg/kg tea tree oil; TTO, tea tree oil*.

### Immune Response

The effect of TTO on the immune response is shown in Table 4. TTO immunomodulation effects were quantified in 28- and 56-days serum using immunonephelometry. On day 28, the diets supplemented with TTO increased (*P* < 0.05) IgG and IgM, but the levels of IgA did not alter (*P* > 0.05) compared with the control group. On day 56, TTO supplemented diets had increased (*P* < 0.05) levels of IgG, unlike the levels of IgA and IgM, which did not alter compared with the control groups.

**Table 4:** Effects of tea tree oil on the serum immunity of finishing pigs (*n* = 6).

		**Treatment^1^**					
**Item**	**Time /d**	**CON**	**LTO**	**MTO**	**HTO**	**SEM**	***P*-value**
IgA/(μg/mL)	28	104.26	165.44	151.70	120.34	9.23	0.06
	56	114.90	123.58	116.85	126.10	4.68	0.83
IgG/(μg/mL)	28	384.90^b^	490.47^a^	575.80^a^	463.71^a^	18.61	<0.001
	56	273.04^b^	371.49^a^	407.97^a^	366.82^a^	17.24	0.03
IgM/(μg/mL)	28	82.05^b^	116.96^a^	122.48^a^	89.44^b^	5.14	0.003
	56	83.07	91.18	89.31	87.62	3.4	0.87

*^a, b^Means in the same row with different superscripts differ significantly for treatment effect. ^1^CON (control), pigs receiving a control diet; LTO (low tea tree oil supplemented treatment), pigs receiving a control diet supplemented with 100 mg/kg tea tree oil; MTO (middle tea tree oil supplemented treatment), pigs receiving a control diet supplemented with 200 mg/kg tea tree oil; HTO (high tea tree oil supplemented treatment), pigs receiving a control diet supplemented with 300 mg/kg tea tree oil; TTO, tea tree oil*.

### Serum Biochemical Indexes

The effect of TTO on serum biochemical indexes of finishing pigs are illustrated in Table 5. In 28 days, TTO supplementation increased (*P* < 0.05) serum ALP levels and decreased (*P* < 0.05) serum AST concentration, relative to control. In 56 days, serum AST concentration decreased (*P* < 0.05) with TTO supplementation compared with control groups.

**Table 5:** Effects of tea tree oil on the serum enzyme of finishing pigs (*n* = 6).

		**Treatment^1^**					
**Item**	**Time /d**	**CON**	**LTO**	**MTO**	**HTO**	**SEM**	***P*-value**
ALT/(U/L)	28	54.50	51.50	47.67	47.50	1.08	0.052
	56	50.91	56.17	51.33	54.33	1.58	0.63
AST/(U/L)	28	47.00^a^	44.17^ab^	40.83^bc^	36.83^c^	1.35	0.04
	56	49.00^a^	54.67^a^	39.50^b^	36.00^b^	2.08	0.001
ALP/(U/L)	28	70.66^b^	73.17^b^	93.33^a^	85.93^a^	2.52	<0.001
	56	130.00	148.50	143.17	138.00	4.90	0.61

*>^a, b, c^Means in the same row with different superscripts differ significantly for treatment effect. ^1^CON (control), pigs receiving a control diet; LTO (low tea tree oil supplemented treatment), pigs receiving a control diet supplemented with 100 mg/kg tea tree oil; MTO (middle tea tree oil supplemented treatment), pigs receiving a control diet supplemented with 200 mg/kg tea tree oil; HTO (high tea tree oil supplemented treatment), pigs receiving a control diet supplemented with 300 mg/kg tea tree oil; TTO, tea tree oil*.

### Antioxidant Status

As shown in Table 6, MTO and HTO supplementation decreased (*P* < 0.05) MDA and increased (*P* < 0.05) GSH-Px activity in serum, and GSH-Px activity increased with 200 mg/kg TTO supplementation in LD muscle compared with the control groups.

**Table 6:** Effects of tea tree oil supplementation on antioxidant activity in finishing pigs (*n* = 6).

		**Treatment^1^**					
**Sites**	**Symbol**	**CON**	**LTO**	**MTO**	**HTO**	**SEM**	***P*-value**
Serum	MDA(nmol/ml)	2.97^a^	3.08^a^	1.99^b^	1.93^b^	0.17	0.014
	CAT(U/ml)	5.68	4.76	5.68	5.04	0.17	0.13
	T-SOD(U/ml)	114.52	114.84	120.52	126.57	4.02	0.71
	GSH-PX(U/ml)	879.02^b^	889.69^b^	963.49^a^	966.73^a^	10.8	<0.001

*^a, b^Means in the same row with different superscripts differ significantly for treatment effect. ^1^CON (control), pigs receiving a control diet; LTO (low tea tree oil supplemented treatment), pigs receiving a control diet supplemented with 100 mg/kg tea tree oil; MTO (middle tea tree oil supplemented treatment), pigs receiving a control diet supplemented with 200mg/kg tea tree oil; HTO (high tea tree oil supplemented treatment), pigs receiving a control diet supplemented with 300 mg/kg tea tree oil; TTO, tea tree oil. MDA, malondialdehyde; CAT, catalase; T-SOD, total superoxide dismutase; GSH-Px, glutathione peroxidase*.

### Carcass Traits and Meat Quality

The effect of TTO on carcass traits and meat quality of finishing pigs are listed in Table 7. Compared with the control group, the liver weight was increased (*P* < 0.05) in MTO and HTO groups. As regards to meat quality, there were the highest pH 45 min value and intramuscular fat and lowest cooking loss, cooking loss, and shear force in the MTO group (*P* < 0.05).

**Table 7:** Effects of tea tree oil on the carcass traits and meat quality of finishing pigs (*n*=6)^1^.

		**Treatment^2^**					
**Item**	**CON**	**LTO**	**MTO**	**HTO**	**SEM**	***P*-value**
**Carcass traits**
Liver weight (kg)	1.58^b^	1.64^b^	1.87^a^	1.95^a^	0.05	0.003
Heart weight (kg)	0.41	0.44	0.42	0.40	0.01	0.623
Spleen weight (kg)	0.34	0.38	0.37	0.40	0.01	0.173
Pancreas weight (kg)	0.20	0.17	0.18	0.15	0.01	0.646
**Meat quality**
pH_24h_	5.68^b^	5.70^b^	5.77^a^	5.72^b^	0.01	0.003
L*	37.30	37.61	37.56	36.65	0.53	0.92
a*	5.24	4.39	5.28	3.69	0.30	0.21
b*	3.00	3.67	3.57	2.89	0.16	0.23
Drip loss (%)	43.99	42.00	42.77	41.47	0.45	0.22
Cooking loss (%)	43.61^a^	37.56^c^	41.27^b^	41.28^b^	0.52	<0.001
Shear force (kg)	2.82^a^	2.45^c^	2.50^bc^	2.69^ab^	0.045	0.005
Intramuscular fat (%)	2.70^b^	3.15^a^	3.15^a^	2.77^ab^	0.07	0.04

*^1^Part of data has been used in our previous study (13). ^a, b, c^Means in the same row with different superscripts differ significantly for the treatment effect. ^2^CON (control), pigs receiving a control diet; LTO (low tea tree oil supplemented treatment), pigs receiving a control diet supplemented with 100 mg/kg tea tree oil; MTO (middle tea tree oil supplemented treatment), pigs receiving a control diet supplemented with 200 mg/kg tea tree oil; HTO (high tea tree oil supplemented treatment), pigs receiving a control diet supplemented with 300 mg/kg tea tree oil; TTO, tea tree oil*.

L*, lightness; a*, redness; b*, yellowness.

### Fatty Acid Composition in LD Muscle

The effect of TTO on the fatty acid composition and content in LD muscle of finishing pigs are summarized in Table 8. The concentration of C12:0 and C16:0 was decreased (*P* < 0.05) in MTO compared with the control group. Diets supplemented with TTO had increased (*P* < 0.05) C18:2n6t concentration relative to the control group.

**Table 8:** Effects of TTO supplementation on fatty acids composition and content in Longissimus dorsi muscle in finishing pigs (*n* = 6).

		**Treatment^1^**					
**Item**	**CON**	**LTO**	**MTO**	**HTO**	**SEM**	***P*-value**
C10:0	0.12	0.09	0.12	0.14	0.01	0.14
C12:0	0.12^a^	0.10^ab^	0.07^b^	0.13^a^	0.04	0.02
C14:0	1.08	1.06	1.12	1.13	0.02	0.46
C16:0	22.77^a^	22.06^ab^	21.83^b^	22.07^ab^	0.13	0.04
C16:1	2.90	3.06	2.61	3.02	0.07	0.08
C18:0	9.37	9.97	9.40	11.34	0.31	0.08
C18:1	41.94	41.57	41.77	38.96	0.50	0.11
C18:2n6t	3.63^b^	4.06^a^	4.08^a^	4.06^a^	0.05	<0.001
C18:2n6c	12.70	12.27	13.26	12.26	0.18	0.16
C20:1	0.53	0.44	0.43	0.80	0.06	0.10
C20:4	1.94	1.96	1.93	1.92	0.10	0.99
C22:4	0.49	0.35	0.39	0.45	0.03	0.54
SFA^2^	33.47	33.29	32.54	34.81	0.31	0.06
MUFA^3^	45.37	45.07	44.82	42.78	0.47	0.20
PUFA^4^	18.77	18.64	19.66	18.70	0.18	0.17
PUFA/SFA	0.56^b^	0.56^b^	0.60^a^	0.54^b^	0.01	0.003

*^a, b^Means in the same row with different superscripts differ significantly for treatment effect. ^1^CON (control), pigs receiving a control diet; LTO (low tea tree oil supplemented treatment), pigs receiving a control diet supplemented with 100 mg/kg tea tree oil; MTO (middle tea tree oil supplemented treatment), pigs receiving a control diet supplemented with 200 mg/kg tea tree oil; HTO (high tea tree oil supplemented treatment), pigs receiving a control diet supplemented with 300 mg/kg tea tree oil; TTO, tea tree oil. ^2^SFA = C10:0+C12:0+C14:0 + C16:0 + C17:0 + C18:0; ^3^MUFA = C16:1 + C18:1+ C20:1; ^4^PUFA = C18:2n6t + C18:2n6c + C20:4 + C22:4*.

### The Genes Expression of H-FABP, CAPN1, CAST, and HAL in LD Muscle

The effects of TTO supplementation on the gene expression of H-FABP, CAPN1, CAST, and HAL in the LD muscle in finishing pigs are shown in Table 9. The mRNA expression of H-FABP and CAPN1 increased by supplementation with 200 mg/kg TTO compared with the control group.

**Table 9:** Effects of TTO supplementation on the genes expression of H-FABP, CAPN1, CAST, and HAL in Longissimus dorsi muscle in the finishing pigs (*n* = 6).

		**Treatment^1^**					
**Symbol**	**Control**	**LTO**	**MTO**	**HTO**	**SEM**	***P*-value**
*H-FABP*	1.00^b^	1.08^b^	1.37^a^	0.60^c^	0.06	<0.001
*CAPN1*	1.00^b^	0.96^b^	1.29^a^	1.34^a^	0.05	0.001
*CAST*	1.01	1.01	1.10	1.20	0.09	0.89
*HAL*	1.00	1.09	1.14	1.06	0.09	0.96

*^a, b, c^Means in the same row with different superscripts differ significantly for treatment effect. ^1^CON(control), pigs receiving a control diet; LTO (low tea tree oil supplemented treatment), pigs receiving a control diet supplemented with 100 mg/kg tea tree oil; MTO (middle tea tree oil supplemented treatment), pigs receiving a control diet supplemented with 200 mg/kg tea tree oil; HTO (high tea tree oil supplemented treatment), pigs receiving a control diet supplemented with 300 mg/kg tea tree oil; TTO, tea tree oil*.

## Discussion

Tea tree oil extract from the leaves of M. alternifolia exhibits anti-inflammatory, antioxidant, and anti-bacterial properties (9). Dong et al. (14) reported that TTO has the potential to replace the usage of antibiotics in weanling pigs. In our current study, dietary supplementation with TTO improves meat quality by increasing intramuscular fat content and tenderness (13). Meat is a primary source of essential fatty acids for human health. It has been shown that a positive effect exists between TTO and lipid metabolism (16). However, there is limited information in the mechanism regarding the effect of TTO on growth performance, meat quality, serum biochemical indices, and antioxidant capacity in the finishing pigs. Hence, the present study investigated the effect of TTO on growth performance, meat quality, serum biochemical indices, and antioxidant capacity in finishing pigs.

Our previous studies have demonstrated that TTO could improve the growth performance of finishing pigs (13). In this study, we further investigated the mechanisms that the effect of TTO on growth performance. GH–IGF axis components play important roles in regulating growth in the finishing pigs. GH is a vital gene regulating the growth and development of pigs. Skarphedinsson et al. (17) reported that GH can promote protein synthesis, while protein degradation rate was not affected. IGF-I can stimulate cell proliferation, differentiation, and other cellular functions in different tissues (18). An IGF-I level is a valuable tool for estimating growth rate and is positively correlated with growth rate in cattle and pigs (18). The positive correlation between IGF-I expression in the liver and growth performance has been demonstrated (19). Our results showed that TTO supplementation increased the mRNA expression of GH and IGF-I in the liver and LD muscle, indicating that protein metabolism might be improved due to TTO supplementation. In addition, IGF-I and IGF-II have similar functions, which can promote growth performance. In this study, the expression of IGF-II was not affected in the liver, LD muscle, and back fat tissues by supplementation with TTO, which is consistent with the study that IGF-II plays a vital role in the embryonic development rather than in postnatal development (20). MSTN, a member of the transforming growth factor type b (TGFβ) super-family, is a negative regulator of muscle growth (21). This research showed that the mRNA expression of MSTN was decreased in the liver and LD muscle by TTO supplementation.

Hormones associated with the hypothalamic-pituitary-adrenal axis are the main effectors for translating stress into a physiological action. These hormones include the cortisol-containing glucocorticoid group, which are widely regarded as regulators of immune function (22–24). In addition, the ability of a TTO supplemented diet to mount an innate immune response is well documented (8). Serum IgA, IgG, and IgM levels positively correlate with body immunity. IgG is the primary immunoglobulin that can protect animals against infections by microorganisms (25). Our result showed that supplementation with TTO increased the serum IgG and IgM concentration. Based on this research, supplementation with TTO could enhance the immune response in finishing pigs.

The liver is a digestive gland that secretes bile, participates in the metabolism of protein, sugars, and fat, and has essential functions, including detoxification and provision of immunity (26). The ALT and AST are clinically helpful in evaluating acute hepatocellular injury in viral hepatitis (27). The levels of ALT and AST activities are negatively correlated with the health of hepatocytes. The activity of ALT and AST is at a low level when the liver is in a healthy state, and the activity of ALT and AST is significantly increased when liver cells are damaged (28). The biologically active compounds in TTO also influence liver metabolism (16), as lower activity of AST was determined in blood plasma, which agrees that dietary supplementation with TTO increased the liver weight, indicating improved liver health originating from TTO supplementation.

Reactive oxygen species (ROSs) are produced by cells during normal metabolism (29). However, ROS over-generation in pigs leads to oxidative stress, resulting in reduced immune function and decreased growth performance (30). The antioxidant capacity of the host was evaluated by the determination of related enzymes inhibiting ROS formation, such as MAD, SOD, T-AOC, and GSH-Px. Broiler fed with essential oil in diet exhibited increased T-SOD and T-AOC levels, but down-regulation of MDA concentration has been reported (31, 32). Puvaca et al. (33) reported that a diet supplemented with TTO improves the anti-oxidative ability of laying hens by increasing SOD and GSH-Px activities. Supplementation of animal diets with essential oil to feed can improve the antioxidant status of the animal body and increase meat quality (34). Consistent with previous studies, our result demonstrated that diet supplemented with TTO enhanced GSH-Px levels and decreased MAD levels in serum. Improved oxidative status observed when TTO supplemented diets are fed might be due to its ingredients, such as α-terpinene, terpinen-4-ol, and γ-terpinene, which have enhanced antioxidant abilities described in the previous study (35, 36).

Our previous studies have demonstrated that TTO could improve meat quality (13). Meat is a primary source of fat in human diets. It has been reported that saturated fatty acid (SFA) may increase cholesterol levels and the risk of cardiovascular diseases (37, 38). Previous experiments conducted on TTO supplementation mainly focused on its anti-inflammatory and anti-oxidative ability, although the essential oils have a positive effect on body lipid metabolism (15). The concentration of fatty acids can influence the meat quality and meat products (39). However, limited studies have been conducted to evaluate the effect of TTO supplementation on the fatty acid composition of pork. Therefore, we hypothesized that dietary supplementation with TTO could increase the polyunsaturated fatty acid (PUFA) and SFA concentration in meat. The mass consumption of SFA has been associated with an increased risk of obesity and the inception of other related diseases. Conversely, UFA, especially PUFA, reduced the risk of coronary heart disease (40). Fatty acid composition positively correlates with meat quality by determining the nutritional value of muscle and oxidative stability. The level of fatty acid saturation can affect the degree of fat firmness, consequently influencing the quality and acceptability of meat products (39). Therefore, appropriate levels of SFA and PUFA should be maintained to ensure superior meat quality. In the current study, diets supplemented with TTO had decreased SFA (lauric acid (C:12:0) and palmitic acid (C:16:0) and increased PUFA (mainly methyl linolelaidate (C18:2n6t) levels in meat, which confirmed our hypothesis. H-FABP is involved in the intracellular targeting of fatty acids and facilitates the transport of fatty acids from the membrane to the sites of fatty acid oxidation or esterification into TAG or phospholipids (41, 42). H-FABP, first discovered in the heart, has been associated with the intramuscular fat content of pigs. Meanwhile, the mRNA expression of H-FABP affects the intramuscular fat content of pigs (43). In the present study, diet supplemented with TTO increased the mRNA expression of H-FABP. It has been reported that mRNA expression of H-FABP was positively correlated with intramuscular fat content (44), which agrees with our results. Previous researches indicated that higher intramuscular fat content could lead to improvements of flavor, marbling score, and tenderness of pork, and the quality and content of intramuscular fat content could be influenced by various factors including nutrition levels, nutrients, market weight, age, and animal breed (45–47). In addition, supplementation with perilla seed extract significantly increased the intramuscular fat content of fattening cattle's longissimus muscle (48). Tenderness is another index of meat quality. The extent of protein proteolysis is the main factor in determining tenderness (49). Calpains belong to the protease family and play a vital role in meat quality. Moreover, there is a positive association between calpain activity and meat quality (50). It has been demonstrated that calpain 1 is mainly responsible for myofibrillar protein degradation in the skeletal muscle (51). The CAST act as a calpain-specific endogenous inhibitor (52). In the present study, supplementation with TTO increased the mRNA expression of calpain 1, but the expression of CAST was not affected in the LD muscle.

In conclusion, we provide significant evidence for the effect of TTO on growth performance, meat quality, serum biochemical indices, and antioxidant capacity in the finishing pigs. Supplementation with TTO improve growth performance through modulating the expression genes of GH, IGF-I, and MSTN. Supplementing this ingredient into the finishing pigs' feed improved the meat quality by increasing the ratio of PUFA/SFA, modulating the expression genes associating with meat quality, and improving intramuscular fat content. Simultaneously, TTO can improve antioxidant capacity and body immunity in the finishing pigs. Therefore, TTO is recommended for supplementation in the diet of the finishing pigs.

## Data Availability Statement

The original contributions presented in the study are included in the article/Supplementary Material, further inquiries can be directed to the corresponding author.

## Ethics Statement

The animal study was reviewed and approved by Animal Care and Use Committee of Yangzhou University, Yangzhou, China. Written informed consent was obtained from the owners for the participation of their animals in this study.

## Author Contributions

TY performed experiment work, analyzed the data, and wrote the manuscript. FF also performed experiment work. MJ and XM revised the manuscript. KZ and OD writing—reviewing and editing. XZ and YH provide the support of TTO extract. GZ contributed to the experimental idea. All authors contributed to the article and approved the submitted version.

## Funding

This study was supported by the National Natural Science Foundation of China (No. 32002200), the Research Project of Natural Science Foundation of the Jiangsu Province (BK20190898), the China Agriculture Research System of MOF and MARA, the Jiangsu Province industry-university-research cooperation-prospective Joint Research Project (BY2016069-12), and Excellent Doctoral Dissertation Fund of the Yangzhou University.

## Conflict of Interest

XZ was employed by Wuxi Chenfang Biotechnology Co., Ltd. The remaining authors declare that the research was conducted in the absence of any commercial or financial relationships that could be construed as a potential conflict of interest.

## Publisher's Note

All claims expressed in this article are solely those of the authors and do not necessarily represent those of their affiliated organizations, or those of the publisher, the editors and the reviewers. Any product that may be evaluated in this article, or claim that may be made by its manufacturer, is not guaranteed or endorsed by the publisher.

## References

[1] Ni JQ Shi C Liu SL Richert BT Vonderohe CE Radcliffe JS. Effects of antibiotic-free pig rearing on ammonia emissions from five pairs of swine rooms in a wean-to-finish experiment. Environ Int. (2019) 131:104931. 10.1016/j.envint.2019.104931 31319291

[2] Henchion M McCarthy M Resconi VC Troy D. Meat consumption: trends and quality matters. Meat Sci. (2014) 98:561–8. 10.1016/j.meatsci.2014.06.007 25060586

[3] Kristensen L Stoier S Wurtz J Hinrichsen L. Trends in meat science and technology: the future looks bright, but the journey will be long. Meat Sci. (2014) 98:322–9. 10.1016/j.meatsci.2014.06.023 25028094

[4] Zeng WC Wen WT Deng Y Tian YY Sun HH Sun Q. Chinese ethnic meat products: continuity and development. Meat Sci. (2016) 120:37–46. 10.1016/j.meatsci.2016.04.007 27091319

[5] Maron DF Smith TJS Nachman KE. Restrictions on antimicrobial use in food animal production: an international regulatory and economic survey. Glob Health. (2013) 9:48. 10.1186/1744-8603-9-48 24131666 PMC3853314

[6] Li HG Zhao JS Deng W Li K Liu HW. Effects of chlorogenic acid-enriched extract from Eucommia ulmoides Oliver leaf on growth performance and quality and oxidative status of meat in finishing pigs fed diets containing fresh or oxidized corn oil. J Anim Physiol. (2020) 104:1116–25. 10.1111/jpn.13267 31802552

[7] Sailer R Berger T Reichling J Harkenthal M. Pharmaceutical and medicinal aspects of Australian tea tree oil. Phytomedicine. (1998) 5:489–95. 10.1016/S0944-7113(98)80048-2 23196035

[8] Zhan K Yang T Feng B Zhu X Chen Y Huo Y. The protective roles of tea tree oil extracts in bovine mammary epithelial cells and polymorphonuclear leukocytes. J Anim Sci Biotechnol. (2020) 11:62. 10.1186/s40104-020-00468-9 32549980 PMC7294674

[9] Low WL Martin C Hill DJ Kenward MA. Antimicrobial efficacy of liposome-encapsulated silver ions and tea tree oil against pseudomonas aeruginosa, staphylococcus aureus and candida albicans. Lett Appl Microbiol. (2013) 57:33–9. 10.1111/lam.12082 23581401

[10] Lee SY Chen PY Lin JC Kirkby NS Ou CH Chang TC. Melaleuca alternifolia induces heme oxygenase-1 expression in murine raw264.7 cells through activation of the Nrf2-ARE pathway. Am J Chin Med. (2017) 45:1631–48. 10.1142/S0192415X17500884 29121804

[11] Souza CF Baldissera MD Silva LD Geihs MA Baldisserotto B. Is monoterpene terpinen-4-ol the compound responsible for the anesthetic and antioxidant activity of Melaleuca alternifolia essential oil (tea tree oil) in silver catfish? Aquaculture. (2018) 486:217–23. 10.1016/j.aquaculture.2017.12.025

[12] Wang L Zhang Y Liu L Huang F Dong B. Effects of three-layer encapsulated tea tree oil on growth performance, antioxidant capacity, and intestinal microbiota of weaned pigs. Front Vet Sci. (2021) 8:789225. 10.3389/fvets.2021.789225 34926648 PMC8674471

[13] Feng FF Fang W Wang SN Zhan K Wei ZW Zhao GQ. Effects of tea tree oil on growth performance, organ indexes, carcass characteristics and meat quality of finishing pigs. Chin J Anim Nutr. (2017) 29:3620–6. 10.3969/j.issn.1006-267x.2017.10.024

[14] Dong L Liu J Zhong ZX Wang SN Wang HR Huo YJ. Dietary tea tree oil supplementation improves the intestinal mucosal immunity of weanling piglets. Anim Feed Sci Tech. (2019) 255:114209. 10.1016/j.anifeedsci.2019.114209

[15] Acamovic T Brooker JD. Biochemistry of plant secondary metabolites and their effects in animals. P Nutr Soc. (2005) 64:403–12. 10.1079/PNS2005449 16048675

[16] Yang T Ma X Jiang M Cheng Z Datsomor O Zhao G. The role of tea tree oil in alleviating palmitic acid-induced lipid accumulation in bovine hepatocytes. Front Vet Sci. (2021) 8:814840. 10.3389/fvets.2021.814840 35127885 PMC8814581

[17] Skarphedinsson O Power DM Ingleton PM. Separation of rainbow trout (Salmo gairdneri) growth hormone by gel electrophoresis. Gen Comp Endocrinol. (1990) 80:393–8. 10.1016/0016-6480(90)90188-R 2127033

[18] Liu GM Wei Y Wang ZS Wu D Zhou AG Liu GL. Effects of herbal extract supplementation on growth performance and insulin-like growth factor (IGF)-I system in finishing pigs. J Anim Feed Sci. (2008) 17:538–47. 10.22358/jafs/66681/2008

[19] Pell JM Saunders JC Gilmour RS. Differential regulation of transcription initiation from insulin-like growth factor-I (IGF-I) leader exons and of tissue IGF-I expression in response to changed growth hormone and nutritional status in sheep. Endocrinology. (1993) 132:1797–807. 10.1210/endo.132.4.8462477 8462477

[20] Gerrard DE Okamura CS Ranalletta MA Grant AL. Developmental expression and location of IGF-I and IGF-II mRNA and protein in skeletal muscle. J Anim Sci. (1998) 76:1004–11. 10.2527/1998.7641004x 9581923

[21] McPherron AC Lawler AM Lee SJ. Regulation of skeletal muscle mass in mice by a new TGF-beta superfamily member. Nature. (1997) 387:83–90. 10.1038/387083a0 9139826

[22] Dantzer R Kelley KW. Stress and immunity. Life Sci. (1989) 44:1995–2008 10.1016/0024-3205(89)90345-7 2568569

[23] Brown-Borg HM Klemcke HG Blecha F. Lymphocyte profilerative responses in neonatal pigs with high or low plasma cortisol concentration after stress induced by restraint. Am J Vet Res. (1993) 54:2015–20 8116931

[24] Maule AG VanderKooi SP. Stress-induced immune-endocrine interaction. In: Balm P editor. Stress Physiology. Sheffield: Sheffield Academic Press Ltd. (1999). p. 205-45.

[25] Gomez GG Phillips O Goforth RA. Effect of immunoglobulin source on survival, growth, and hematological and immunological variables in pigs. J Anim Sci. (1998) 76:1–7. 10.2527/1998.7611 9464877

[26] Hou Q Qian Z Wu P Shen M Li L Zhao W. 1-Deoxynojirimycin from mulberry leaves changes gut digestion and microbiota composition in geese. Poult Sci. (2020) 99:5858–66. 10.1016/j.psj.2020.07.048 33142503 PMC7647860

[27] Cohen JA Kaplan MM. The SGOT/SGPT ratio–an indicator of alcoholic liver disease. Dig Dis Sci. (1979) 24:835–8. 10.1007/BF01324898 520102

[28] Sui YN Ma TT Chen ZY. Effects of dietary calcium N-hydroxymethylmethionine on serum biochemical indices of lactation cows. Chin J Agr University. (2017) 22:94–101. 10.11841/j.issn.1007-4333.2017.03.1231439996

[29] Yu BP. Cellular defenses against damage from reactive oxygen species. Physiol Rev. (1994) 74:139–62. 10.1152/physrev.1994.74.1.139 8295932

[30] Lauridsen C Hojsgaard S Sorensen MT. Influence of dietary rapeseed oil, vitamin E, and copper on the performance and the antioxidative and oxidative status of pigs. J Anim Sci. (1999) 77:906–16. 10.2527/1999.774906x 10328356

[31] Faix S Faixova Z Placha I Koppel J. Effect of cinnamomum zeylanicum essential oil on antioxidative status in broiler chickens. Acta Vet Brno. (2009) 78:411–7. 10.2754/avb200978030411

[32] Habibi R Sadeghi G Karimi A. Effect of different concentrations of ginger root powder and its essential oil on growth performance, serum metabolites and antioxidant status in broiler chicks under heat stress. Brit Poultry Sci. (2014) 55:228–37. 10.1080/00071668.2014.887830 24697550

[33] Puvaca N Lika E Cocoli S Kika TS Bursic V Vukovic G. Use of tea tree essential oil (Melaleuca alternifolia) in laying hen's nutrition on performance and egg fatty acid profile as a promising sustainable organic agricultural tool. Sustain Basel. (2020) 12:3420. 10.3390/su12083420

[34] Jiang H Zhan WQ Liu X Jiang SX. Chemical composition and antioxidant activity of the essential oil from oxytropis falcate bunge. J Essent Oil Res. (2009) 21:300–2. 10.1080/10412905.2009.9700176

[35] Ruberto G Baratta MT. Antioxidant activity of selected essential oil components in two lipid model systems. Food Chem. (2000) 69:167–74. 10.1016/S0308-8146(99)00247-2

[36] Kim HJ Chen F Wu CQ Wang X Chung HY Jin ZY. Evaluation of antioxidant activity of Australian tea tree (Melaleuca alternifolia) oil and its components. J Agr Food Chem. (2004) 52:2849–54. 10.1021/jf035377d 15137824

[37] Siri-Tarino PW Chiu S Bergeron N Krauss RM. Saturated fats versus polyunsaturated fats versus carbohydrates for cardiovascular disease prevention and treatment. Annu Rev Nutr. (2015) 35:517–43. 10.1146/annurev-nutr-071714-034449 26185980 PMC4744652

[38] Ruiz-Nunez B Dijck-Brouwer DAJ Muskiet FAJ. The relation of saturated fatty acids with low-grade inflammation and cardiovascular disease. J Nutr Biochemist. (2016) 36:1–20. 10.1016/j.jnutbio.2015.12.007 27692243

[39] Perry D Nicholls PJ Thompson JM. The effect of sire breed on the melting point and fatty acid composition of subcutaneous fat in steers. J Anim Sci. (1998) 76:87–95. 10.2527/1998.76187x 9464888

[40] Picklo MJ Idso J Seeger DR Aukema HM Murphy EJ. Comparative effects of high oleic acid vs high mixed saturated fatty acid obesogenic diets upon PUFA metabolism in mice. Prostaglandins Leukot Essent Fatty Acids. (2017) 119:25–37. 10.1016/j.plefa.2017.03.001 28410667

[41] Krag MB Gormsen LC Guo Z Christiansen JS Jensen MD Nielsen S. Growth hormone-induced insulin resistance is associated with increased intramyocellular triglyceride content but unaltered VLDL-triglyceride kinetics. Am J Physiol Endocrinol Metab. (2007) 292:E920–7. 10.1152/ajpendo.00374.2006 17132823

[42] Tyra M Ropka-Molik K. Effect of the FABP3 and LEPR gene polymorphisms and expression levels on intramuscular fat (IMF) content and fat cover degree in pigs. Livest Sci. (2011) 142:114–20. 10.1016/j.livsci.2011.07.003

[43] Gerbens F van Erp AJM Harders FL Verburg FJ Meuwissen THE Veerkamp JH. Effect of genetic variants of the heart fatty acid-binding protein gene on intramuscular fat and performance traits in pigs. J Anim Sci. (1999) 77:846–52. 10.2527/1999.774846x 10328348

[44] Gerbens F Verburg FJ Van Moerkerk HTB Engel B Buist W Veerkamp JH. Associations of heart and adipocyte fatty acid-binding protein gene expression with intramuscular fat content in pigs. J Anim Sci. (2001) 79:347–54. 10.2527/2001.792347x 11219443

[45] Wang Z. Effects of N-Carbamylglutamate on growth performance nutrient digestibility and serum free amino acids of weaned piglets. Chin J Anim Nutr. (2010) 22:1012–8.

[46] Park G Moon S Ko Y Ha J Lee J Chang H Joo S. Influence of slaughter weight and sex on yield and quality grades of Hanwoo (Korean native cattle) carcasses. J Anim Sci. (2002) 80:129–36. 10.2527/2002.801129x 11831510

[47] Teye G Sheard P Whittington F Nute G Stewart A Wood J. Influence of dietary oils and protein level on pork quality. 1. effects on muscle fatty acid composition, carcass, meat and eating quality. Meat Sci. (2006) 73:157–65. 10.1016/j.meatsci.2005.11.010 22062065

[48] Zhang H. Effects of perilla seed extract on intramuscular fat deposition in fattening cattle. Chin J Anim Nutr. (2019) 31:1897–903.

[49] Koohmaraie M Geesink GH. Contribution of postmortem muscle biochemistry to the delivery of consistent meat quality with particular focus on the calpain system. Meat Sci. (2006) 74:34–43. 10.1016/j.meatsci.2006.04.025 22062714

[50] Sentandreu MA Coulis G Ouali A. Role of muscle endopeptidases and their inhibitors in meat tenderness. Trends Food Sci Tech. (2002) 13:400–21. 10.1016/S0924-2244(02)00188-7

[51] Geesink GH Kuchay S Chishti AH Koohmaraie M. Micro-calpain is essential for postmortem proteolysis of muscle proteins. J Anim Sci. (2006) 84:2834–40. 10.2527/jas.2006-122 16971586

[52] Chen L Feng XC Zhang WG Xu XL Zhou GH. Effects of Inhibitors on the synergistic interaction between calpain and caspase-3 during post-mortem aging of chicken meat. J Agr Food Chem. (2012) 60:8465–72. 10.1021/jf300062n 22720745

