# Supplementary material for: Effect of the Tea Tree Oil on Growth Performance, Meat Quality, Serum Biochemical Indices, and Antioxidant Capacity in Finishing Pigs
Source: Front Vet Sci. 2022 Jun 24;9:916625. doi: 10.3389/fvets.2022.916625 (PMC9263609; doi:10.3389/fvets.2022.916625)
Supplement: Supplementary file 1 [file Table_1.DOCX]

**Supplementary Table 1.** Effect of TTO on the growth performance of finishing pigs

|  | Treatment^1^ | | | |  |  |
| --- | --- | --- | --- | --- | --- | --- |
| Items | CON | LTO | MTO | HTO | SEM | *P*-value |
| Initial weight/kg | 67.93 | 68.31 | 68.25 | 68.00 | 0.09 | 0.48 |
| Final weight/kg | 114.29^b^ | 119.04^a^ | 119.81^a^ | 119.56^a^ | 0.74 | 0.007 |
| ADG/(g/d) | 827.75^b^ | 905.88^a^ | 920.76^a^ | 920.76^a^ | 13.04 | 0.01 |
| ADFI/(kg/d) | 2.95 | 2.91 | 3.02 | 3.04 | 0.03 | 0.34 |
| F/G | 3.58^a^ | 3.21^b^ | 3.28^b^ | 3.30^b^ | 0.05 | 0.047 |

^1^ Part of data has been used in our previous study (13).

^a, b, c^ Means in the same row with different superscripts differ significantly for treatment effect.

^2^ CON (control), pigs receiving a control diet; LTO (low tea tree oil supplemented treatment), pigs receiving a control diet supplemented with 100 mg/kg tea tree oil; MTO (middle tea tree oil supplemented treatment), pigs receiving a control diet supplemented with 200mg/kg tea tree oil; HTO (high tea tree oil supplemented treatment), pigs receiving a control diet supplemented with 300 mg/kg tea tree oil; TTO, tea tree oil.
